# Evaluating the Supplementary Role of Photogrammetry in Insect Taxonomy: Applications and Limitations of 3D Scanning Technology

**DOI:** 10.1002/ece3.71651

**Published:** 2025-08-06

**Authors:** Cameron J. Peacock, William Romeu‐Evans, Simon J. Goodman, Christopher Hassall

**Affiliations:** ^1^ School of Natural Sciences, Faculty of Science and Engineering, University of Manchester Manchester UK; ^2^ School of Biology, Faculty of Biological Sciences University of Leeds Leeds UK

**Keywords:** diagnostics, entomology, identification, methodology, modelling, morphology, photogrammetry, taxonomy

## Abstract

The integration of high‐resolution 3D photogrammetry in insect taxonomy offers potential enhancements to traditional classification methods, particularly in educational and resource‐limited settings. This study assesses the Artec Micro scanner's capability to capture detailed external phenotypic features of insects across a size spectrum from 1.63 to 33.49 mm. Ten samples from unique species were mounted and scanned. The model outputs were evaluated against an identification key, which compiled diagnostic features for the specimens from the wider literature, to describe the specimens to the lowest taxonomic level possible. The results showed that six of the ten specimens could be identified to species level using the scans. Threshold values for body length and width were 10.7 and 4.4 mm, respectively. Below these body dimensions, important diagnostic features of specimens could not be resolved reliably. This result outlines the limitations of this technology and highlights the supportive role that this approach can provide when integrated with traditional taxonomic methods. This approach opens up novel applications for species identification and data sharing among taxonomists, international field research, conservation efforts and entomological outreach. However, the limitations of this approach to taxonomic identification must be considered depending upon the size of the specimen and its diagnostic features. Future developments could enhance this technology's application in routine taxonomic work, particularly through integration with artificial intelligence platforms.

## Graphical abstract

Our study presents a cost‐effective 3D scanning approach to enhance taxonomic identification, particularly for archival and educational applications. By allowing the scanning of pinned specimens without damage, this method supplements traditional taxonomy while promoting accessibility and conservation efforts.

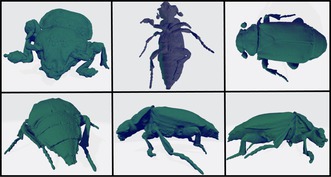

## 1 Introduction

The current number of documented insect species is estimated at over one million, suggesting that only 15% to 20% of insect species have been classified (Foottit and Adler 2009). Insects play a pivotal role in global ecosystems, acting as food sources (Tallamy and Shriver 2021), mediating ecosystem services such as pollination and nutrient recycling (Schowalter et al. 2018), as well as some being disease vectors and parasites (Lounibos 2002). The sheer diversity of insects has required the development of extensive and detailed species identification and description techniques, with an emphasis on rapid and effective identification (Guerra‐García et al. 2008). Despite the importance of taxonomic tools and skills to biodiversity research, concerns have been raised regarding the future of species identification due to loss of taxonomic expertise as experienced researchers retire, and low training rates of new specialists, resulting in a decreasing capacity for species identification (Lee 2000). Various pressures such as climate change and human activity are exacerbating these concerns by driving many species towards extinction at a rapid rate, creating a biodiversity crisis, which requires extensive monitoring (Bellard et al. 2012).

### 1.1 Benefits of 3D Photogrammetry

Traditional taxonomic methods, while precise, are often resource‐intensive and require specialised expertise that is becoming scarce. Three‐dimensional photogrammetric scanning offers a promising enhancement to contemporary taxonomic practice (e.g., SEM and Micro‐CT scanners; see Table 1 for a detailed comparison) due to advantages such as its low cost, ability to rapidly generate high‐fidelity 3D models with little preparation requirements and its user‐friendly programming requiring little training. These benefits were highlighted in a paper by Nguyen et al. (2013) who demonstrated the applications of 3D models in rapid identification of invasive insect species, citing the speed and accuracy of specimen classification as a key advantage over conventional 2D imaging methods. Another key benefit of this approach is the scanners portability, which reduces the need for specimen preservation and transportation off site. This further reduces costs and streamlines the identification process, with potential to help mitigate some of the issues around erosion of taxonomic expertise by facilitating access to specimens and training, and by opening new possibilities for artificial intelligence supported taxonomic identification.

**TABLE 1:** A comparison of scanner attributes between the Artec 3D Micro Scanner, Zeiss EVO SEM and Zeiss Micro‐CT. Information sourced from personal communication (see Appendix S3).

Scanner attributes	Artec 3D scanner	Zeiss EVO SEM	Zeiss Micro‐CT
Produce 3D model	Yes	No	Yes
Resolve wings	Yes	Yes	Yes, but challenging
Resolve dense areas of hair	No	Yes	Yes
Needs extensive training	No	Yes	Yes (including software training)
Cost per sample	Negligible	£20	£150+
Scanning time per sample	20 min	30 min	40 min–5 h
Purchase cost	£32,000	£114,000–£170,000	£240,000–£490,000
Resolution	0.029 mm (however, scans show a mean minimum width for resolution of 0.35 mm)	50–100 nm (can be improved to 1 nm—10 nm Hitachi SUB8230 but costs more per sample)	0.25–150 μm (can be limited for insect scanning due to small elements and wing movement due to their flexibility)

### 1.2 Comparison of 2D and 3D Imaging

More traditional approaches to taxonomic identification, particularly in the fields of entomology and ecology, are macro and microphotography. A study by McCullough et al. (2013) highlighted the applications of macrophotography for capturing high‐resolution images of fast‐moving insects, to monitor biodiversity and allow for insect identification. The benefits of this approach include the ability to obtain images of specimens without the need for capture or euthanasia. This research also demonstrated the advantages of creating virtual collections for areas such as education of undergraduates and outreach. However, the issues surrounding macrophotography typically arise when trying to distinguish between species with very similar exterior morphologies (Martínez‐Gamba and Rincón 2020). Microphotography is also a widely used approach for taxonomic identification due to its ability to resolve diagnostic characters at a higher resolution for a substantially reduced cost when compared to 3D scanning approaches. This technique is mainly used for documenting external features, with it providing vital specimen information for identification, such as colouration, degree of sclerotisation and transparency (Wipfler et al. 2016). The major drawback of 3D photogrammetry when compared to macro and microphotography is the lack of colour within the models, which is often pivotal for insect classification. However, the benefits gained from generating a 360° model, which can be spatially manipulated, as well as allowing for accurate measurements of morphological traits, such as surface area and length, give credence to this approach. Additionally, advances in 3D technology are being made to allow for colour capture during the scanning process (Nguyen et al. 2017; Qian et al. 2019). Despite this, capturing colour currently requires increased scanning time, as well as additional complexity and workload maintaining updates to software and hardware. Despite this, we suggest the combination of the capabilities of 3D photogrammetry provide a useful complement to traditional imaging methodologies to create the most advantageous identification protocol.

### 1.3 Applications of 3D Modelling

The technique of 3D scanning has been applied to various areas of taxonomic research, including virtual palaeontology for fossil reconstruction (Bartolini‐Lucenti et al. 2021; Theodorou et al. 2018), detailed identification and cataloguing of novel arthropod species (Hita Garcia et al. 2017), and, in institutions that act as reference archives, improving data availability and removing the need for transporting physical specimens through digitisation in natural history collections (Plum and Labonte 2021; Rybenská and Borůvková 2020). Additionally, a study by Castro et al. (2022) demonstrated a significant increase in learning performance in zoology students taught using 3D models, highlighting the potential for integration of these models into educational institutions to aid in taxonomic training, particularly for institutions, which are distant from large biological collections such as natural history museums. Here, we evaluate the practical taxonomic ability of photogrammetric 3D scanning technology to resolve characters important for taxonomic identification of insects with varying sizes and surface features, focusing on its applications in educational and archival settings. Although it is not a stand‐alone substitute for detailed morphological or molecular analysis, this technology could play a crucial role in modern taxonomy by supporting species identification and facilitating the training of new taxonomists.

## 2 Methods

### 2.1 Specimen Selection and Sampling

We examined 10 specimens from different species, which were selected to give a range of different body sizes and morphological characteristics. Specimens were scanned using an Artec Micro scanner (Artec3D; Luxembourg) with a precision of 10 μm and a resolution of 29 μm. The individuals chosen represented species from Coleoptera, Lepidoptera, Diptera and Orthoptera (Appendix S1). Specimens ranged in body length from 1.63 to 33.49 mm and possessed unique diagnostic traits to allow for identification, such as wing morphology, body colour and pattern, etc. Bat fly (Nycteribiidae spp.) and bat bug specimens (Cimicidae spp) (*n* = 2), originally sampled in Baja California, Mexico (Najera‐Cortazar et al. 2023), were obtained from a collection held at the University of Leeds. The remaining specimens were sourced from a routine insect monitoring programme operating at Hatfield Moors, UK (53.533° N, −0.978° E).

### 2.2 Specimen Preparation

Samples had been preserved in 70% ethanol and therefore required rehydration using acetone and a heat lamp (Grissell and Schauff 1995). Based on the scanner manufacturer's recommendations, each specimen was then sprayed with a thin layer of Aesub blue scanning spray to create a matte finish and prevent glare during scanning. Each specimen was mounted on a needle and secured on the scanning plate.

### 2.3 Scanning Procedure and Model Generation

The Artec Micro scanner employs an internal calibration routine, and scans were performed using the manufacturer‐recommended settings for small, complex specimens (the small complex fine setting in the Artec Studio 3D software). No external calibration standards were required. Once a scan was initiated, the scanner arm rotated around the sample, taking an initial 30 scans from a range of angles under the default settings. Additional scans could be added to enhance model completeness if required. The scans are automatically compiled into a 3D model for each specimen, which was then refined using the sharp fusion and hole filling tools in the Artec Studio program to generate a watertight model (a complete model with no unfilled areas or holes). The models were then used to determine body length, width, wing length and volume for each specimen.

### 2.4 Identification

A taxonomic identification key was generated by compiling a range of diagnostic features collected from pre‐established taxonomic keys (see Appendix S2). This key, along with the 3D generated models, was then used to determine the taxonomic level to which each of the 10 specimens could be identified. Each specimen was then designated a taxonomic score based on this. A score of zero meant unidentifiable and scores ranging from one to five represented class, order, family, genus and species, respectively.

### 2.5 Data Analysis

**FIGURE 1:**
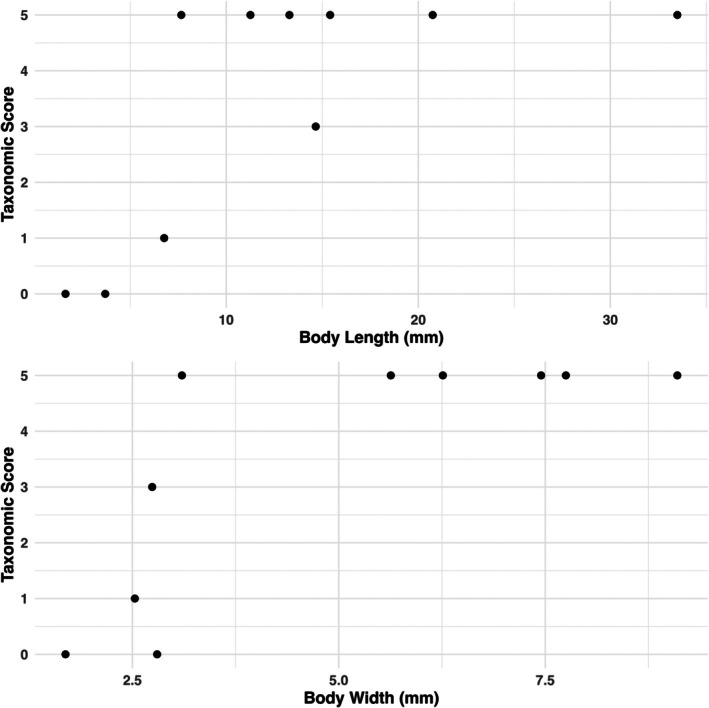
The relationship between two metrics of body size (graph A representing body length and graph B representing body width) and the taxonomic score achieved for each specimen. The taxonomic score is ranked from 0 to 5, with zero being unidentifiable and 1, 2, 3, 4 and 5 corresponding to class, order, family, genus and species, respectively.

Body size measurements were plotted against taxonomic scores for each specimen to estimate resolution threshold value of each trait (Figure 1). For the Artec scanner to be an effective tool for taxonomic identification we suggest that family should be the minimum level of classification achievable, as below this point diagnostic features become more diverse and difficult to distinguish (e.g., hair presence/length, wing venation). Family‐level identification is also the standard taxonomic resolution in freshwater biomonitoring. Therefore, the point where the fit line intercepts the taxonomic score of 3 was used to determine the threshold value.

## 3 Results

**FIGURE 2:**
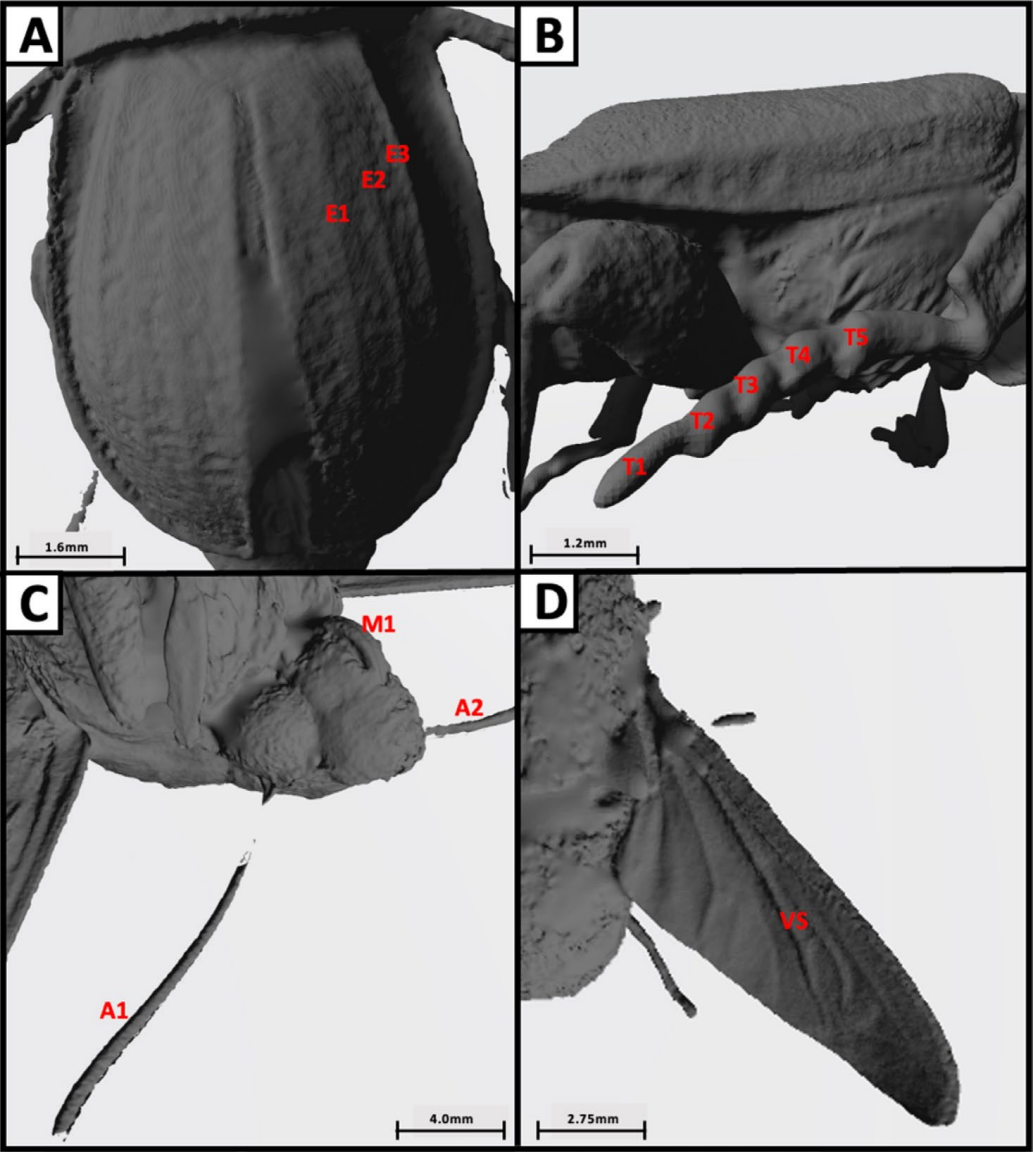
A collection of images taken of the Artec 3D scans of various specimens, highlighting various important diagnostic features for the identification of different taxonomic groups. (A) Depicts the raised longitudinal lines on the elytra of *Phosphuga atrata* (with three of these on the right side of the elytra being labelled E1, E2, E3). (B) Depicts the segments of the tarsi of *Nicrophorus interruptus* (labelled T1 to T5), used to identify *Nicrophorus*. (C) Depicts the antennae and mouthpart of *Deilephila elpenor* (labelled M1 for mouthpart and A1/A2 for the antennae), used to identify Lepidoptera. (D) Depicts the vena spuria (labelled VS) present in the centre of the wing of *Eristalis pertinax*, used to identify Syrphidae. Scale bar is attributed to each photo as scale varies between scan captures.

**FIGURE 3:**
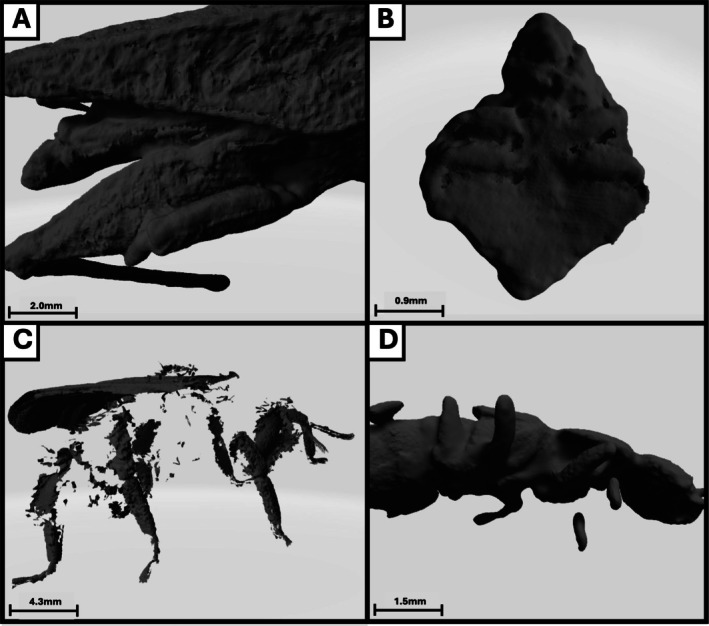
A collection of images taken of the Artec 3D scans of various specimens, highlighting limitations of this approach when trying to resolve various taxonomic groups. (A) Depicts the fusion of limbs of *Tetrix undulata*. (B) Depicts the inability to resolve features of a small bat bug specimen. (C) Depicts the inability to resolve areas of densely packed hair of a *Bombus* species. (D) Depicts the incomplete resolution of the lower legs of *Quedius xanthopus*. Scale bar is attributed to each photo as scale varies between scan captures.

It was found that seven of the total 10 specimens could be identified to family level or below, using diagnostic features compiled from numerous sources. The bat bug and bat fly ectoparasites, which had an average length of 2.67 mm, average width of 2.25 mm and average volume of 2.33 mm^3^ could not be identified to any taxonomic level. The third smallest specimen, *Sarcophaga* spp., could only be identified to the level of class (Insecta). This specimen had a body length of 6.77 mm, body width of 2.53 mm and a volume of 29.39 mm^3^. The ranges for body size metrics for the remaining seven specimens were 7.66 to 33.49 mm for body length, 2.74 to 9.10 mm for body width and 51.11 to 1394.22 mm^3^ for volume. Estimated resolution threshold values were 10.7 mm for body length, 4.4 mm for body width, 4.2 for surface area to volume ratio and 112 mm^3^ for volume. Finally, the minimum width resolved for each specimen was averaged, giving a mean of 0.35 mm, suggesting that any feature with a width lower than this is unlikely to be resolved. The process of using the 3D models to identify specimens to different taxonomic levels provided insight into the various diagnostic features, which could be successfully resolved (Figure 2) and those that failed (Figure 3).

## 4 Discussion

This study was carried out to evaluate the application of 3D scanning for taxonomic identification of insects and to determine the threshold values for body size where the resolution of important diagnostic features becomes limited. The Artec 3D scanner is capable of effectively generating 3D models that can aid in the taxonomic classification of insects with body lengths and widths exceeding 10.7 and 4.4 mm, respectively. This included resolving a range of diagnostic features needed for the taxonomic identification of species from various groups, including Lepidoptera, Diptera and Coleoptera (see Figure 2). However, the resolution constraints emphasise the current technological limits in capturing a multitude of fine details that are crucial for lower taxonomic classification of other groups such as Hymenoptera and Araneae. Despite these challenges, 3D photogrammetry scanning holds substantial promise for enhancing taxonomic training and public engagement, which is significant as this can aid in tackling the issue of the erosion of taxonomic expertise as a result of the retirement of taxonomists and low training rates of new specialists. This technology could serve as a valuable adjunct to traditional methods, allowing for the use of interactive and detailed models in educational settings and to enhance data sharing between institutions through digitised archiving of specimens. This study identified key limitations of using 3D modelling for arthropod taxonomic identification, particularly focusing on body size and the resolution of fine diagnostic features. Additional issues with this approach included the fusing of limbs and the inability to resolve areas of dense hairs (e.g., in *Bombus* spp.). This aligns with results obtained by Doan and Nguyen (2024), whose novel 3D photogrammetry approach to arthropod identification also struggled with thin structures such as the legs and antennae, as well as setae. However, Plum and Labonte (2021) detailed that these issues can be remedied by increasing the number of camera positions during a scan, although this does result in longer processing times. Additionally, their 3D scanning approach (scAnt) was capable of resolving morphological structures as fine as 30 μm, with the paper stating that the size of the scanned animal was a key factor as absolute resolution scales with animal size. Future research should aim to investigate the furthest limits of this technology by including a wider range of species from various families. By increasing the number of unique individuals, a database of diagnostic features could be created to inform future applications of this technology for taxonomic identification. Further detail could be added using textures captured with high‐resolution photographs.

**FIGURE 4:**
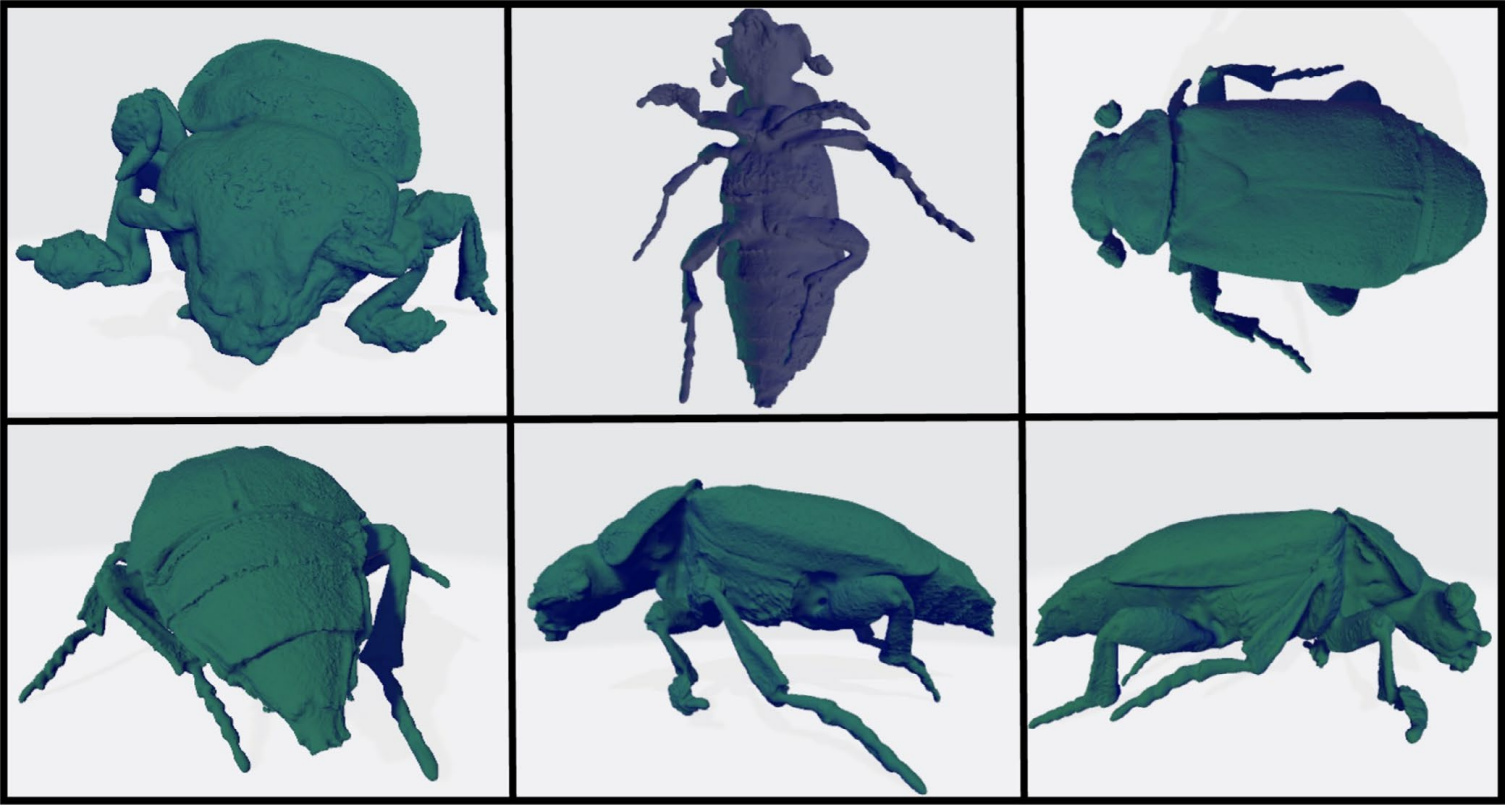
A collection of images taken of the Artec 3D scan of *Nicrophorus interruptus*, highlighting a subset of the various orientations that the model can be observed from. Viewing the model in the program Artec studio allows for a 360° view of the specimen, with the ability to zoom into important diagnostic features and manipulate the orientation of the model.

In comparison to other high‐resolution imaging technologies, such as scanning electron microscopy and Micro‐CT scanning, the Artec 3D scanner provides many benefits, which aid its supplementary use within taxonomy. These include the ability to manipulate the models' orientation to allow for viewing of the specimen from various angles (360° viewing; Figure 4), the ability to zoom into small diagnostic features and a toolkit within the software, which allows the user to take accurate length, volume and surface area measurements. The value of these measurements was highlighted in a study by Qian et al. (2019) who looked at a new 3D photogrammetry approach to scanning *Coleoptera*, finding that curvature, volume and length values could have applications for morphometric research into the relationship between compound eye function and insect phototaxis. Additionally, the 3D photogrammetric scanners involve lower purchasing and running costs, shorter processing times and require less training than other conventional high‐resolution scanning techniques. Finally, the technology's compatibility with metal pins enables scanning of preprepared specimens, such as those in museum archives, without removing the pins, which is a requirement often incompatible with Micro‐CT scanning. This feature avoids the need for alternative fixatives and reduces the risk of specimen damage.

Next steps would be blind trials with taxonomists, asking groups to identify specimen sets using 3D models without prior knowledge. This would help refine scanner protocols for taxonomic identification and demonstrate how this method can increase capacity for species identification. Additionally, recent developments of new 3D scanning technology such as scAnt, an open‐source cost‐effective platform, provide the opportunity for comparative empirical studies to examine the efficacy of different technologies in terms of taxonomic identification (Plum and Labonte 2021). If these technologies are applied on a wider scale and incorporated into the existing taxonomic toolkit, this approach has the potential to enhance novel species discovery, support conservation efforts involving rare or undescribed species and promote public engagement around insect biodiversity topics.

## 5 Conclusion

This study demonstrates that photogrammetric 3D scanning can effectively capture important diagnostic external features in larger insect specimens, providing a viable complementary tool for taxonomy, education and museum digitisation. While the technology has clear limitations for smaller or hair‐covered specimens, it is an affordable and portable tool for use in 3D modelling and has the ability to generate manipulatable 3D models, which offer meaningful benefits for a range of entomological applications. This includes morphology measurements such as limb length, body surface area and volume. As scanning and processing technology continues to evolve, future research should focus on improving resolution, comparing available systems in taxonomic workflows and eventually incorporating 3D photogrammetric approaches alongside more traditional visualisation methods, such as microphotography and SEM.

## Author Contributions

**Cameron J. Peacock:** conceptualization (equal), data curation (lead), formal analysis (lead), investigation (lead), methodology (equal), writing – original draft (lead), writing – review and editing (lead). **William Romeu‐Evans:** data curation (supporting), formal analysis (supporting), investigation (supporting), methodology (supporting), supervision (supporting), writing – original draft (supporting), writing – review and editing (supporting). **Simon J. Goodman:** conceptualization (lead), data curation (supporting), investigation (supporting), methodology (equal), supervision (lead), writing – original draft (supporting), writing – review and editing (equal). **Christopher Hassall:** supervision (supporting), validation (supporting), writing – review and editing (equal).

## Conflicts of Interest

The authors declare no conflicts of interest.

## Supporting information


Data S1.

**Appendix S1.** The identification key used for each specimen, including the significant diagnostic features used in the wider literature to identify various taxonomic groups.
**Appendix S2.** A list of sources used to generate the taxonomic identification key found in Appendix S1. This includes the author(s), date of publication, name of article, hyperlink to the source, and the date that we accessed the resource.
**Appendix S3.** A record of correspondence to gather information used in Table 1, including their position, the line of contact used and the date of correspondence.

## Data Availability

All raw data utilised to form conclusions in this study is present in Data S1.
