# Supplementary material for: Evaluating the Supplementary Role of Photogrammetry in Insect Taxonomy: Applications and Limitations of 3D Scanning Technology
Source: Ecol Evol. 2025 Aug 6;15(8):e71651. doi: 10.1002/ece3.71651 (PMC12326419; doi:10.1002/ece3.71651)
Supplement: Supplementary file 1 — Data S1. [file ECE3-15-e71651-s002.docx]

Raw Data

| Species | Body  Width | Body  Length | Volume | Surface  area | Wing  length | Wing  width | SA/V | Taxon  Score | Max resolved feature |
| --- | --- | --- | --- | --- | --- | --- | --- | --- | --- |
| T. undulata | 3.1 | 13.29 | 74.27 | 208.57 | n/a | n/a | 2.81 | 5 | 0.27 |
| C. septempunctata | 7.75 | 7.66 | 94.22 | 268.27 | 3.74 | 7.98 | 2.85 | 5 | 0.31 |
| N. interruptus | 7.45 | 20.75 | 422.75 | 582.65 | n/a | n/a | 1.38 | 5 | 0.28 |
| E. pertinax | 5.63 | 15.41 | 291.56 | 549.53 | 3.83 | 10.88 | 1.88 | 5 | 0.46 |
| P. atrata | 6.26 | 11.26 | 236.17 | 507.42 | n/a | n/a | 2.15 | 5 | 0.33 |
| S. argyrostoma | 2.53 | 6.77 | 29.39 | 117.48 | 2.02 | 6.2 | 4.00 | 1 | 0.44 |
| Q. xanthopus | 2.74 | 14.66 | 51.11 | 135.08 | n/a | n/a | 2.64 | 3 | 0.23 |
| D. elpenor | 9.1 | 33.49 | 1394.22 | 2750.57 | 16.35 | 32.6 | 1.97 | 5 | 0.57 |
| Batbug spp. | 2.8 | 3.7 | 1.98 | 18.1 | n/a | n/a | 9.14 | 0 | 0.29 |
| Batfly spp. | 1.69 | 1.63 | 2.68 | 21.09 | n/a | n/a | 7.87 | 0 | 0.35 |
