# Supplementary material for: Evaluating the Supplementary Role of Photogrammetry in Insect Taxonomy: Applications and Limitations of 3D Scanning Technology
Source: Ecol Evol. 2025 Aug 6;15(8):e71651. doi: 10.1002/ece3.71651 (PMC12326419; doi:10.1002/ece3.71651)
Supplement: Supplementary file 2 — Appendix S1. The identification key used for each specimen, including the significant diagnostic features used in the wider literature to identify various taxonomic groups. Appendix S2. A list of sources used to generate the taxonomic identification key found in Appendix S1. This includes the author(s), date of publication, name of article, hyperlink to the source, and the date that we accessed the resource. Appendix S3. A record of correspondence to gather information used in Table 1, including their position, the line of contact used and the date of correspondence. [file ECE3-15-e71651-s001.docx]

Appendix 1

The identification key used for each specimen, including the significant diagnostic features used in the wider literature to identify various taxonomic groups. The sources of these diagnostic features can be found in Appendix 2. The table also includes the taxonomic score achieved for each specimen, with 0 meaning unidentifiable and 1 to 5 corresponding to class, order, family, genus, and species respectively. The body length of each species is also provided.

| **Scientific Name** | **Identification Characters** | **Taxa score**  **(0-5)** | **Body Length**  **(mm)** |
| --- | --- | --- | --- |
| Nycteribiidae spp. | None of the diagnostic features in the literature for identification, such as flattened body, lack of wings and ocelli and leg morphology, could be resolved. | 0 | 1.63 |
| Cimicidae spp. | None of the diagnostic features described in the literature for identification, such as antennae length/ segment number, tarsi morphology, head shape and ocelli presence, length of fringe hairs on protonum, could be resolved. | 0 | 3.70 |
| *Sarcophaga spp.* | 1. Jointed legs, hard cuticle, six legs, head/thorax/abdomen – **Insecta**  2. Has fully developed wings, one pair of well-developed wings with hindwings reduced to small knobs behind the forewings – **Diptera**  3. Not metallic and shiny, typically black, and grey. Dorsum of thorax often striped and abdomen chequered **– Sarcophagidae**  4. Terminal abdominal segment usually red, less than 10mm long, three dark stripes on thorax**,** large red eyes**- *Sarcophaga***  5. Species are often difficult to distinguish, with the most reliable method involving examination of the genitalia under microscope. | 1 | 6.77 |
| *Coccinella septempunctata* | 1. Jointed legs, hard cuticle, six legs, head/thorax/abdomen – **Insecta**  2. Fully developed wings, visible mouthparts, forewings are shield-like and lack visible veins, forming a straight line where they meet dorsally – **Coleoptera**  3. 1- 7mm in Length, body typically ovate or hemispherical, moderately to strongly convex, often with spots on pronotum and elytra – **Coccinellidae**  4/5. Orange/ red elytra with black spots, seven spots in total (key feature) – ***Coccinella septempunctata*** | 5 | 7.66 |
| *Phosphuga atrata* | 1. Jointed legs, hard cuticle, six legs, head/thorax/abdomen – **Insecta**  2. Fully developed wings, visible mouthparts, forewings are shield-like and lack visible veins, forming a straight line where they meet dorsally – **Coleoptera**  3. Relatively large (9-30mm), antennae widely spaced and insert on the lateral side of the head, flattened dorsally, tarsi of all six legs have 5 segments, antennae clubbed or wider at the tip – **Silphidae**  4/5. Uniform colouration (mainly dark), antennae expanded apically but lack clubbed tip, head elongated, elytra with raised longitudinal lines – ***Phosphuga atrata*** | 5 | 11.26 |
| *Tetrix undulata* | 1. Jointed legs, hard cuticle, six legs, head/thorax/abdomen – **Insecta**  2. Two pairs of developed wings, visible and developed mandible mouthparts, abdomen tip lacks obvious pincers, forewings leathery and veins are visible, body is not flattened and head visible from above, front legs are simple but hind legs are enlarged for jumping – **Orthoptera**  3. Antennae shorter than body, size between 8 and 14 mm, elongated pronotum, brown colouration - **Tetrigidae**  4/5. Pronotum not extending as far as hind knees, strong midline ridge, hind wings shorter than pronotum – ***Tetrix undulata*** | 5 | 13.29 |
| *Quedius xanthopus* | 1. Jointed legs, hard cuticle, six legs, head/thorax/abdomen – **Insecta**  2. Fully developed wings, visible mouthparts, forewings are shield-like and lack visible veins, forming a straight line where they meet dorsally – **Coleoptera**  3. 1 to 24mm in length, number of tarsi can be 3 for all six legs or 5 for all six legs, mainly characterised by short elytra, generally elongated and slender, abdomen is exposed – **Staphylinidae**  4. Segments 1 and 2 of tarsi are broader than long, antennae are filiform (evenly curved segments give an even outline), elytra uniformly punctured – **Quedius** | 3 | 14.66 |
| *Eristalis pertinax* | 1. Jointed legs, hard cuticle, six legs, head/thorax/abdomen – **Insecta**  2. Has fully developed wings, one pair of well-developed wings with hindwings reduced to small knobs behind the forewings – **Diptera**  3. Typically short and segmented antennae, large eyes, presence of vena spuria (chitinized fold in the wing), some species mimic Hymenoptera but lack features such as stingers or two sets of wings – **Syrphidae**  4/5. Front tarsi are orange/yellow in colour, noticeable dark face stripe, curved rear tibia, 13 – 15mm in length, two vertical bands of hairs on the eyes – **Eristalis pertinax** | 5 | 15.41 |
| *Nicrophorus interruptus* | 1. Jointed legs, hard cuticle, six legs, head/thorax/abdomen – **Insecta**  2. Fully developed wings, visible mouthparts, forewings are shield-like and lack visible veins, forming a straight line where they meet dorsally – **Coleoptera**  3. Relatively large (9-30mm), antennae widely spaced and insert on the lateral side of the head, flattened dorsally, tarsi of all six legs have 5 segments, antennae clubbed or wider at the tip – **Silphidae**  4. Antennae knob-like with four segments – ***Nicrophorus***  5. Black with orange/brown markings on elytra, hind tibia straight, club of antenna largely orange, anterior orange elytral markings widely separated – ***Nicrophorus interruptus*** | 5 | 20.75 |
| *Deilephila elpenor* | 1. Jointed legs, hard cuticle, six legs, head/thorax/abdomen – **Insecta**  2. Two pairs of well-developed wings, tube-like mouthparts, scaled wings which are powder-like – **Lepidoptera**  3. Forewing length greater than 1.2cm, forewing long and slender, pointed apically, frenulum well developed, body not excessively hairy – **Sphingidae**  4/5. wingspan 30 – 60mm, colouration includes browns and pinks, with unique banding and stripes being the key identifying feature **– *Deilephila elpenor*** | 5 | 33.49 |

Appendix 2

| Author/Source | Date Created | Title | Source | Access Date |
| --- | --- | --- | --- | --- |
| Hutson | 1984 | Keds, flat-flies and bat-flies. Diptera, Hippoboscidae and Nycteribiidae. | Handbooks for the identification of British Insects, Royal Entomological Society | 20/01/2022 |
| Nabeshima *et al.* | 2020 | Detection and phylogenetic analysis of *Bartonella* species from bat flies on eastern bent-wing bats (*Miniopterus fuliginosus)* in Japan | Comparative Immunology, Microbiology and Infectious Diseases, volume 73 | 20/01/2022 |
| Choate. P. M. | 2010 | Identification Key to the Principal Families of Florida Hemiptera, s.o. Heteroptera | https://entnemdept.ufl.edu/choate/florida_heteroptera_families.pdf | 20/01/2022 |
| Cranshaw *et al.* | 2013 | Bat Bugs, Bed Bugs and Relatives | https://extension.colostate.edu/topic-areas/insects/bat-bugs-bed-bugs-and-relatives-5-574/ | 20/01/2022 |
| Zettler *et al.* | 2016 | To key or not to key: a new key to simplify & improve the accuracy of insect identification | https://online.ucpress.edu/abt/article-abstract/78/8/626/18870/To-Key-or-Not-to-Key-A-New-Key-to-Simplify-amp | 25/01/2022 |
| Azidah *et al.* | 2000 | Identification of the Diadegma species (Hymenoptera: Ichneumonidae, Campopleginae) attacking the diamondback moth, *Plutella xylostella* (lepidoptera: Plutellidae) | https://www.cambridge.org/core/journals/bulletin-of-entomological-research/article/abs/identification-of-the-diadegma-species-hymenoptera-ichneumonidae-campopleginae-attacking-the-diamondback-moth-plutella-xylostella-lepidoptera-plutellidae/565C507930A5511E99A18ACD04DEFE79 | 25/01/2022 |
| Piper, R. | 2020 | Saproxylic beetles | https://www.rosspiper.net/2020/01/10/saproxylic-beetles/ | 26/01/2022 |
| Hackston, M. | 2015 | Key to the British genera of subfamily Staphylininae | https://quelestcetanimal-lagalerie.com/wp-content/uploads/2012/11/Subfamily-Staphylininae-keys-to-UK-genera.pdf | 26/01/2022 |
| UK Beetle Recording |  | Staphylinidae | https://www.coleoptera.org.uk/family/staphylinidae | 26/01/2022 |
| UK Beetle Recording |  | *Coccinella septempunctata* Linnaeus, 1785 | https://www.coleoptera.org.uk/species/coccinella-septempunctata | 27/01/2022 |
| UK Beetle Recording |  | Silphidae | https://www.coleoptera.org.uk/family/silphidae | 27/01/2022 |
| UK Beetles |  | SILPHIDAE Latreille, 1806 | https://www.ukbeetles.co.uk/silphidae | 27/01/2022 |
| UK Beetles |  | STAPHYLINIDAE Latreille, 1802 | https://www.ukbeetles.co.uk/staphylinidae | 27/01/2022 |
| NC STATE: Agriculture and Life Sciences |  | Silphidae | https://genent.cals.ncsu.edu/insect-identification/order-coleoptera/family-silphidae/ | 27/01/2022 |
| Klimaszewski, J. &Watt, J. C. | 1997 | Coleoptera: family-group review and keys to identification | https://www.biotaxa.org/fnz/article/view/fnz.37 | 28/01/2022 |
| UK Beetle Recording |  | Key to British Silphidae (Burying beetles and allies) | https://www.coleoptera.org.uk/sites/www.coleoptera.org.uk/files/Silphidae%20Key_2019%20version.pdf | 28/01/2022 |
| Gurney, M |  | A picture guide to beetle families | https://drive.google.com/file/d/18xOiNh0_1UY7RFnSeLCkwgRoJDU6RvbX/view | 28/01/2022 |
| Eversham, B. & Prunier, F. | 2016 | Identifying grasshoppers, crickets and allies in beds, cambs and northants | https://www.wildlifebcn.org/sites/default/files/2018-06/Orthoptera%20keys%20v2.1%202016.pdf | 01/02/2022 |
| Wildlife Insight |  | Elephant Hawk-moth and caterpillar (*Deilephila elpenor*) | http://www.wildlifeinsight.com/british-moths/elephant-hawk-moth-and-caterpillar-deilephila-elpenor/ | 01/02/2022 |
| Staffordshire Wildlife Trust |  | Elephant hawk-moth | https://www.staffs-wildlife.org.uk/wildlife-explorer/invertebrates/butterflies/elephant-hawk-moth | 01/02/2022 |
| Zoology.ubc |  | Order LEPIDOPTERA (Moths & Butterflies): Key and Description of Families | https://www.zoology.ubc.ca/bclepetal/Order%20Lepidoptera%20et%20al%20Text%20Files/order_lepidoptera.htm | 01/02/2022 |
| NC STATE: Agriculture and Life Sciences |  | Sphingidae | https://genent.cals.ncsu.edu/insect-identification/order-lepidoptera/family-sphingidae/ | 01/02/2022 |
| Van Veen, M. P. | 2010 | Hoverflies of Northwest Europe | https://books.google.co.uk/books?hl=en&lr=&id=7eZ5DwAAQBAJ&oi=fnd&pg=PA1&dq=hoverfly+species+identification&ots=abckvsKtOT&sig=eh1Zai7oA07l93CsIDKGXwtl7po#v=onepage&q=hoverfly%20species%20identification&f=false | 02/02/2022 |
| Nature Guide UK |  | *Eristalis pertinax* | https://sites.google.com/site/natureguideuk/home/hoverflies/eristalis/eristalis-pertinax | 02/02/2022 |
| NC STATE: Agriculture and Life Sciences |  | Syrphidae | https://genent.cals.ncsu.edu/insect-identification/order-diptera/family-syrphidae/ | 02/02/2022 |
| Speight, M. C. D. | 2014 | StN Key for the identification of the genera of european Syrphidae | https://www.biodiversityireland.ie/wordpress/wp-content/uploads/StN-KEY-to-GENERA-2014.pdf | 02/02/2022 |
| Bugguide |  | Species *Eristalis tenax*– Drone Fly | https://bugguide.net/node/view/7183 | 02/02/2022 |
| NatureSpot |  | Common Drone Fly – *Eristalis tenax* | https://www.naturespot.org.uk/species/common-drone-fly | 02/02/2022 |
| UK Beetles |  | *Phosphuga atrata* Linnaeus, 1758 | https://www.ukbeetles.co.uk/silpha-atrata | 04/02/2022 |
| Salnitska, M. & Solodovnikov, A. | 2019 | Rove beetles of the genus Quedius (Coleoptera, Stapylinidae) of Russia a key to species and annotated catalogue | https://core.ac.uk/download/pdf/269320236.pdf | 04/02/2022 |
| Hackston, M. | 2018 | Key for the identification of British Silphidae | https://sites.google.com/view/mikes-insect-keys/mikes-insect-keys/keys-for-the-identification-of-british-beetles-coleoptera/key-for-the-identification-of-british-silphidae | 04/02/2022 |
| Unwin, D. M. | 1981 | A key to the families of British Diptera | https://fsj.field-studies-council.org/media/351875/vol5.3_143_a.pdf | 04/02/2022 |
| Marshall *et al.* | 2007 | Key to Diptera families - adults | https://nasmus.co.za/wp-content/uploads/2019/01/Suricata-4-12-KEY-TO-DIPTERA-FAMILIES-ADULTS-low-resolution_Part1.pdf | 10/02/2022 |
| Whitmore *et al*. | 2020 | Key to adult flesh flies (Diptera: Sarcophagidae) of the British Isles | https://osf.io/vf5r6 | 10/02/2022 |

Appendix 3

| Correspondence with: | Contact Information | Date Contacted | Date Responded |
| --- | --- | --- | --- |
| John Harrington – Facility Manager at Leeds Electron Microscopy and Spectroscopy Centre | Email – j.p.harrington@leeds.ac.uk | 01/03/2022 | 02/03/2022 |
| Alex Chung – Pre-Sales and Marketing Manager at Central Scanning Ltd. | Email – alex@central-scanning.co.uk | 03/03/2022 | 03/03/2022 |
| Vincent Fernandez – X-Ray Micro-CT Laboratory Manager | Email – v.fernandez@nhm.ac.uk | 01/03/2022 | 03/03/2022 |
